# Rapid Recurrence of Giant Cell Tumour of C2 Vertebra After Long-Term Denosumab Following Surgical Resection

**DOI:** 10.7759/cureus.22000

**Published:** 2022-02-07

**Authors:** Kuldeep Bansal, Sumedha Singh, Abhinandan R Mallepally, Pratyush Shahi

**Affiliations:** 1 Orthopedics, Indian Spinal Injuries Center, Delhi, IND; 2 Radiology, Institute of Medical Sciences and SUM Hospital, Siksha 'O' Anusandhan University, Bhubaneswar, IND; 3 Orthopedics, University College of Medical Sciences, Delhi, IND

**Keywords:** c2 vertebra, resection, cervical-spine, recurrence, denosumab, giant cell tumors

## Abstract

A 25-year-old man presented with symptoms of cervical myelopathy for 10 days. Imaging revealed an expansile, lytic lesion involving the C2 vertebra completely and compressing the spinal cord, suggestive of giant cell tumor (GCT). Tumor resection and posterior stabilization from C1-C4 were done. Histopathology confirmed the diagnosis of GCT. The patient was kept on adjuvant Denosumab (D-ab) for two years with no signs of recurrence. However, discontinuation of D-ab therapy led to recurrence of the tumor within three months, which was managed with repeated surgical resection and anterior instrumentation followed by radiotherapy. To the best of our knowledge, this is the first reported case of GCT involving the upper cervical spine with rapid recurrence following the stoppage of D-ab therapy.

## Introduction

A giant cell tumor (GCT) is a locally aggressive tumor. When involving the upper cervical spine, its proximity with critical neurovascular structures complicates surgical resection and increases the requirement of adjuvant therapy like Denosumab (D-ab). Although D-ab is increasingly being utilized in cases of GCT, rapid recurrence following its discontinuation has been previously reported [1]. We report a case of GCT of the C2 vertebra that was managed with surgical resection and long-term D-ab therapy. However, discontinuation of D-ab led to rapid recurrence of the tumor, which required a second surgery followed by radiotherapy. To the best of our knowledge, this is the first reported case of GCT involving the upper cervical spine with rapid recurrence following the stoppage of D-ab therapy.

## Case presentation

A 25-year-old man presented with gait disturbance, urinary retention, and difficulty in fine finger movements for 10 days. This was associated with neck pain and radiculopathy for three months. There was no history of fever, weight loss, or trauma. Local examination revealed tenderness at the upper cervical spine with poor neck holding. Neurological examination revealed the power of grade 4/5 (Medical Research Council, MRC) in bilateral upper and lower limbs. Sensations were reduced below the C4 dermatomal level. Signs of hypertonia (ankle clonus, extensor plantar response, hyperreflexia) were seen in all extremities.

**Figure 1:**
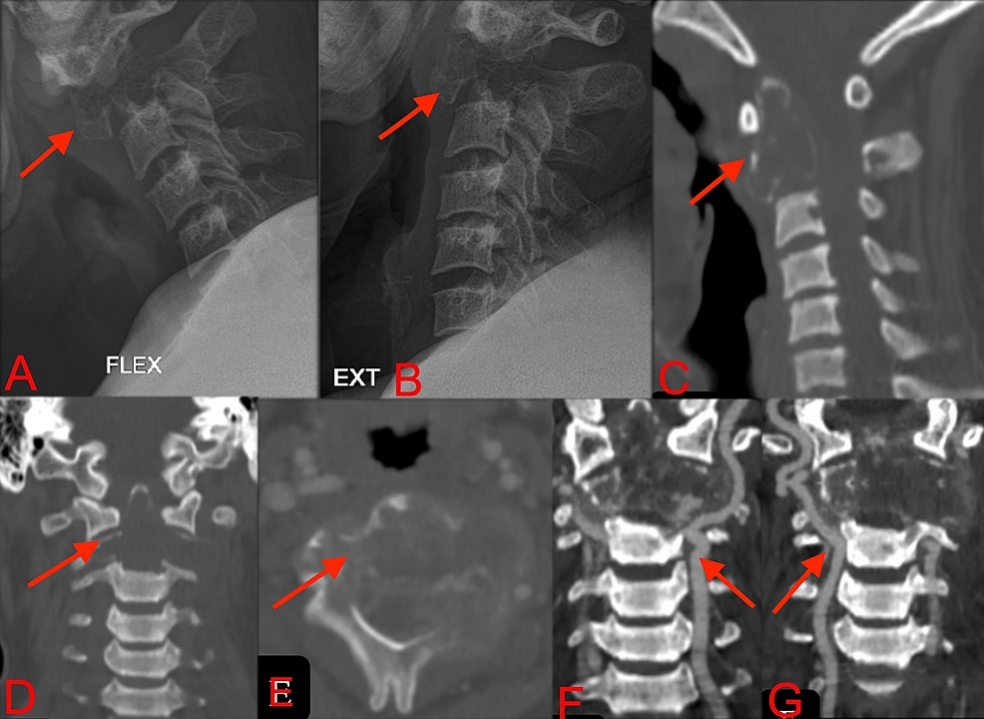
Lateral dynamic radiographs (A, B), CT sections- mid-sagittal (C), coronal (D), axial (E), and CT angiography (F, G) showed an expansile, lytic lesion involving odontoid process and anterior and posterior elements of C2 vertebra with cortical breach and sparing of bilateral vertebral arteries. CT: computerized tomography

**Figure 2:**
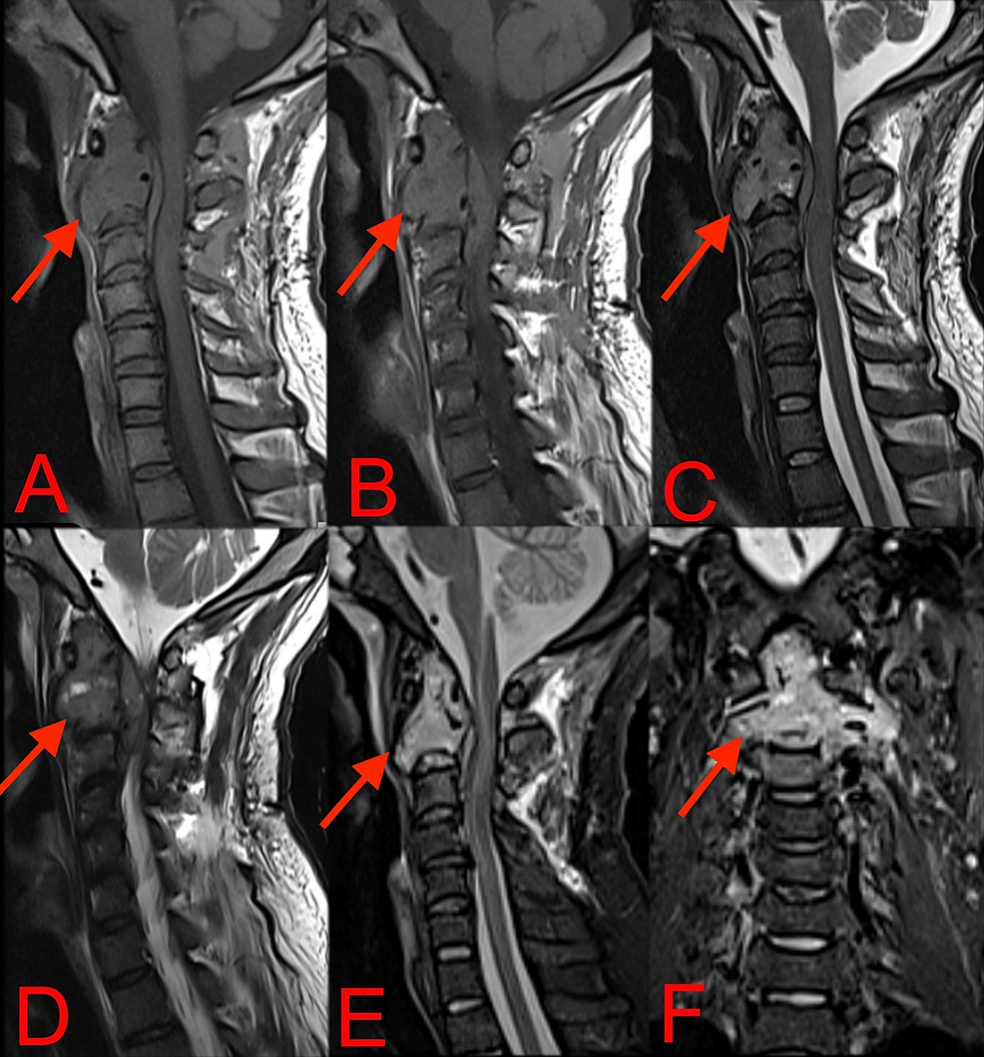
T1 (A, B) and T2 (C, D) weighted mid-sagittal and parasagittal MRI sections and sagittal and coronal STIR (E, F) images showed a lesion which is isointense to muscle on T1 and hyperintense on T2, causing compression of spinal cord with associated cord edema and near-total involvement of the C2 vertebra. MRI: Magnetic Resonance Imaging, STIR: short tau inversion recovery

Plain radiographs and computerized tomography (CT) revealed an expansile, lytic lesion involving the C2 vertebral body with mild instability on flexion and extension (Figure 1). Magnetic Resonance Imaging (MRI) revealed that the lesion involved the C2 vertebra completely, appeared isointense to muscle on T1-weighted and hyperintense on T2-weighted images, and caused mild spinal cord compression at the corresponding level, suggestive of GCT, whereas metastasis generally shows multilevel involvement (Figure 2). Triple phase whole-body nuclear bone scan performed with Technetium 99m-methylene diphosphonate (99mTc MDP) showed a warm spot at the C2 vertebra, and no other high activity lesions were noted in the spine, ruling out the possibility of metastases.

**Figure 3:**
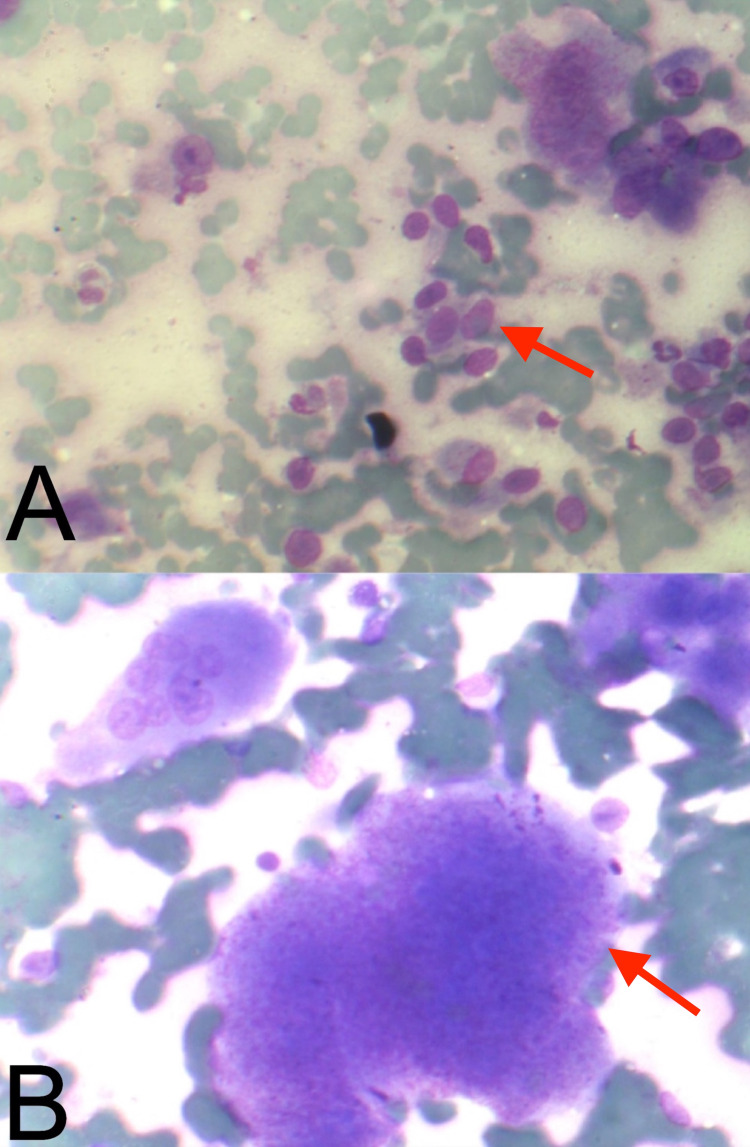
Histopathology showed patchy distribution of monocytes and giant cells among the mature and fused trabecular bone. Giant cells were distributed evenly, including some inflammatory cells and necrotic areas.

**Figure 4:**
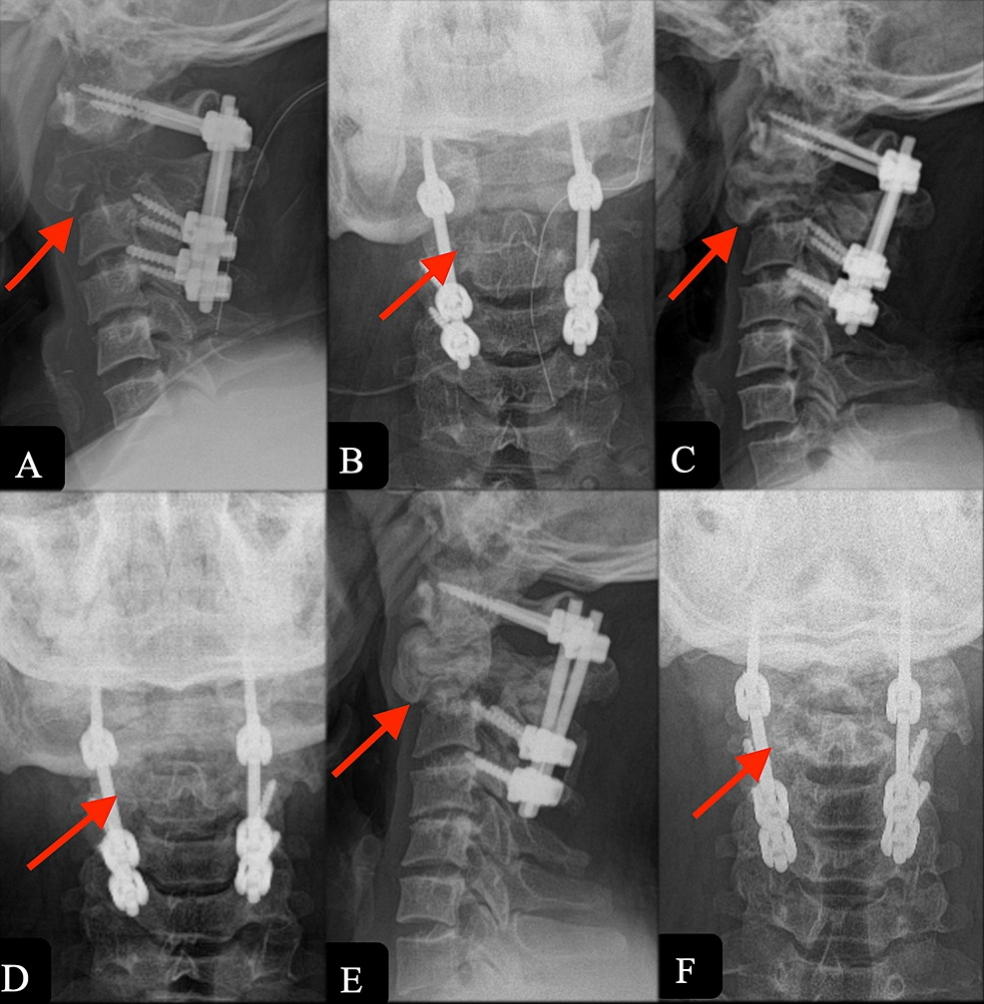
Immediate postoperative lateral and anteroposterior radiographs (A, B) showed tumor resection and posterior fixation from C1-C4. One-year follow-up radiographs (C, D) showed restoration of C2 vertebra with partial sclerosis. Two-year follow-up radiographs (E, F) show complete sclerosis with no evidence of lysis.

Due to extensive anterior involvement and anatomical constraints, tumor resection, C1-C4 stabilization, and biopsy were done through the posterior approach. Histopathological examination showed the presence of multiple prominent osteoclastic giant cells confirming the diagnosis of GCT (Figure 3). Postoperatively, the patient was started on weekly subcutaneous injection of D-ab (120 mg) for three weeks with an additional monthly maintenance dose of 120 mg for 24 months. At the two-year follow-up, there was near-complete remission of the tumor mass and associated symptoms (Figure 4).

**Figure 5:**
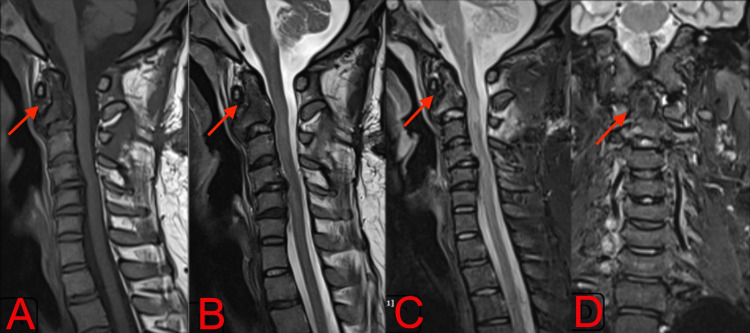
At the two-year follow-up, T1 (A), T2 (B), and STIR (C, D) MRI images showed sclerosis of C2 vertebra with clearance of the spinal canal. MRI: Magnetic Resonance Imaging, STIR: short tau inversion recovery

**Figure 6:**
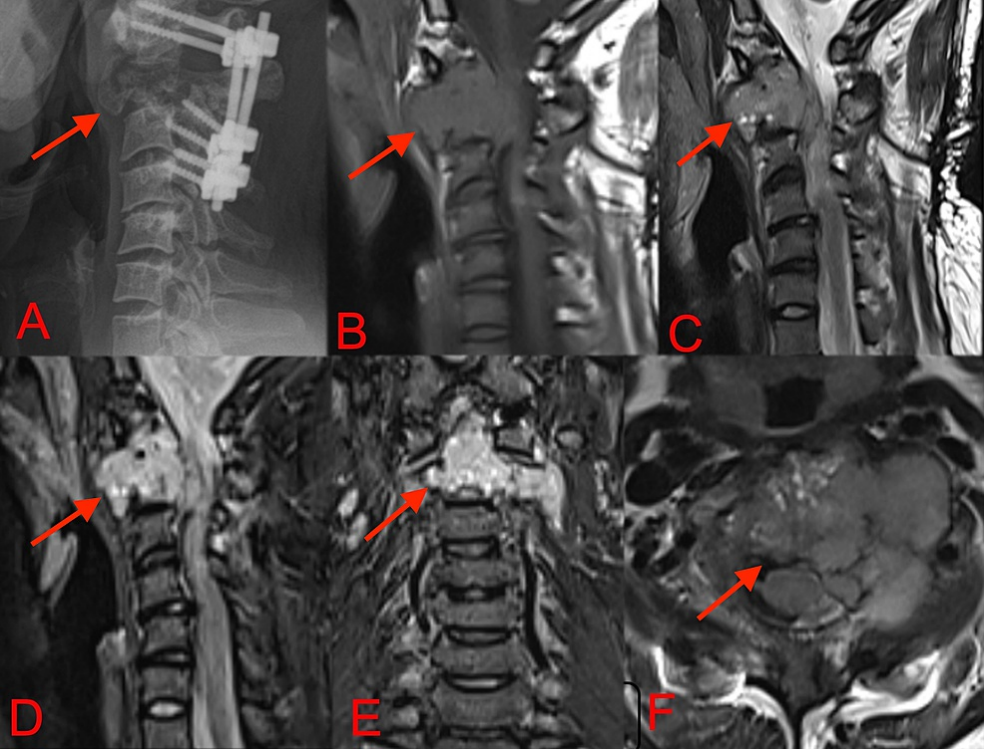
Three months after discontinuation of Denosumab, lateral radiograph (A) and sagittal T1 (B), T2 (C) and STIR (D, E, F) MRI showed a lytic, destructive lesion with similar signal characteristics as previous scan suggesting recurrence of the expansile tumor with destruction of C2 vertebral body and resultant compression of spinal cord with paraspinal soft tissue extension. STIR: short tau inversion recovery, MRI: Magnetic Resonance Imaging

**Figure 7:**
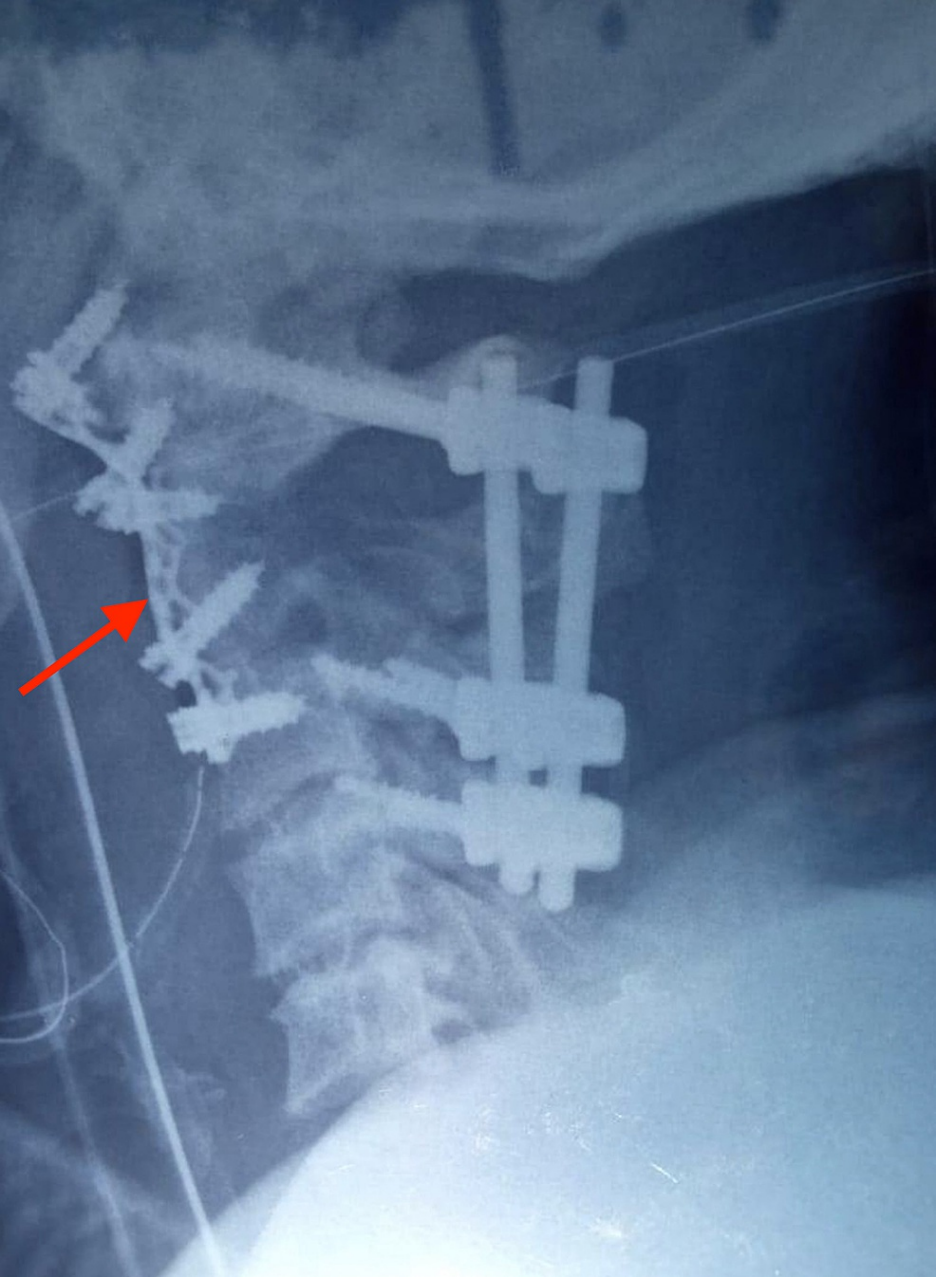
Lateral radiograph after revision surgery showed anterior stabilization spanning C1-C3 and resection of the tumor.

**Figure 8:**
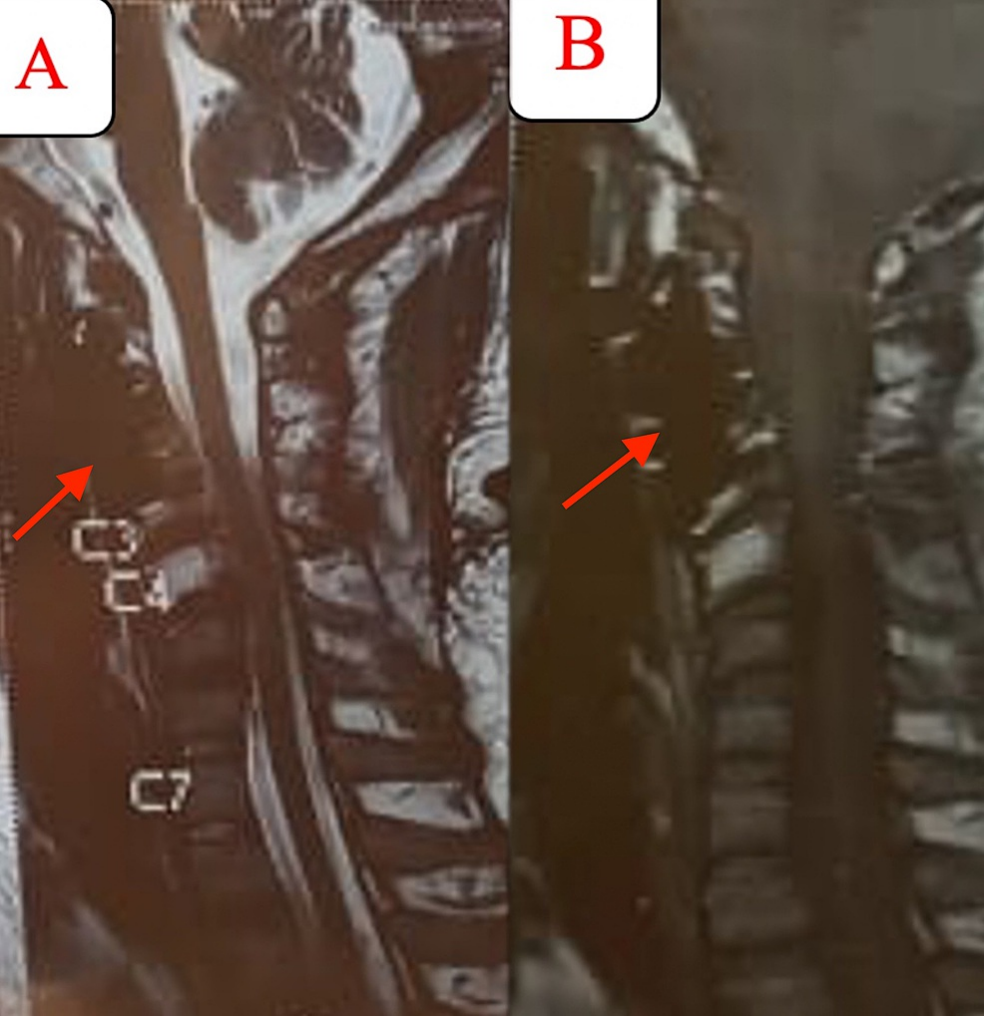
One year after the revision surgery, T2 (A) and T1 (B) sagittal MRI showed resolution of the tumor with spinal canal clearance. MRI: Magnetic Resonance Imaging

However, three months after discontinuation of D-ab therapy at two years, the patient presented with neck pain and bilateral upper limb weakness (power 3/5). Imaging revealed recurrence of the tumor (Figure 5, 6) and was managed with resection and anterior instrumentation (Figure 7) followed by radiotherapy (45 Gy for 25 cycles over five weeks). Volumetric modulated arc therapy (VMAT) on the Elekta Versa HD (Stockholm, Sweden) machine was used for radiotherapy. At the one-year follow-up after the second surgery, there were no clinical or radiological signs of tumor recurrence (Figure 8).

## Discussion

Although GCT is mostly seen in long bones, it can also involve the spine. Primary GCT of the cervical spine accounts for 2%-3% of spine tumors, and GCT in the axial vertebra (C2) is rare [2]. Although regarded as a benign tumor, 5%-10% of GCTs are malignant and aggressive, commonly metastasizing to the lungs [3]. However, when the tumor involves an anatomically complex region, like the C2 vertebra in our case, local aggressiveness poses a higher risk for mortality than distant metastasis.

Patients with spinal GCT generally present with pain, instability, or neural deficits. Treatment options include en-bloc wide resection, intralesional curettage or excision with or without adjuvant techniques, and radiation therapy. The goals of surgery are to preserve function, alleviate pain and prevent local recurrence. Owing to the peculiarity and complexity of the upper cervical spine anatomy, it is challenging to get an adequate tumor-free margin without damaging critical neurovascular structures. Due to hesitant resection, upper cervical GCTs show a higher rate of recurrence. Depending on the tumor’s location, a radical resection procedure such as pan-vertebrectomy may be required, which is associated with significant morbidity [4]. In addition, GCT is a soft and friable tumor; hence en-bloc resection is demanding and less effective due to the risk of tumor cell leakage and seeding. Accuracy of implants and restoration of spinal alignment is also vital to prevent mechanical complications. Although adequate tumor resection and surgical stabilization followed by adjuvant therapy have become the preferred treatment, there is still no consensus regarding the ideal treatment of cervical spine GCT [5].

To prevent recurrence of GCT in the upper cervical spine due to limited resection, adjuvant therapies are required. Potential adjuvant treatments include radiation therapy, embolization, cryosurgery, bisphosphonates, phenol, and D-ab. Radiation can control local growth but is associated with secondary malignant transformation [6]. Long-term responses with phenol are unreliable [7]. D-ab, on the other hand, has been approved by the Food and Drug Administration (FDA) and is being extensively used as adjuvant and neoadjuvant chemotherapy for GCT when the tumor is unresectable or surgical resection has considerable side effects. Table 1 summarizes the previous studies on the utilization of D-ab in the management of cervical GCT [6,8-15].

**Table 1:** Previous studies on utilization of Denosumab in cervical giant cell tumour (GCT).

Author	Age	Sex	Level of cervical spine GCT	Duration	Preoperative administration	Post-operative administration	Response	No. of patients
Mattei et al. (2014) [8]	22y	F	C2	16 months	-	Postoperative Denosumab therapy started 10 months post-surgery	Osteosclerosis seen at three months post- Denosumab	1
Goldschlager et al. (2015) [9]	22y	F	C2	16 months	-	Only arthrodesis without tumor resection followed by Denosumab	Complete	1
Nakazawa et al. (2016) [10]	41y	M	C5	24 months	Conservatively managed with Denosumab	-	Dramatic regression with osteosclerosis	1
Kajiwara et al. (2016) [11]	43y	M	C5	18 months	-	Recurrence at eight months for which Denosumab was given for 18 months	Complete resolution	1
Kumar et al. (2017) [12]	20y	F	C7	Three cycles of Denosumab	Conservatively managed with Denosumab	-	No recurrence	1
Yi et al. (2018) [6]	30y, 33y	F, M	C4, C1-2	Five injections	Conservatively managed with Denosumab	-	No recurrent lesion	2
Law et al. (2018) [13]	53y	M	C3	Once monthly for nine months	Recurrence after six months of Denosumab cessation	Monthly Denosumab for recurrence	Good response	1
Boriani et al. (2019) [14]	28y, 64y, 49y,51y	2M, 2F	C3, C4-5, C5-6, C6	8-20 months	-	Postoperative Denosumab given	Good response in all	4
Heinrich et al. (2019) [15]	15yr	F	C1 lateral mass	Three months	Transoral biopsy with Denosumab for three months	Transoral tumor resection followed by Denosumab	Good	1

The major components of GCT are multinucleate giant cells (osteoclasts) and mononuclear stromal cells. Osteoclasts express receptor activator of nuclear factor-kappa B (RANK), and mononuclear stromal cells express RANK ligand (RANKL). Binding of RANKL to RANK leads to osteoclast activation. D-ab specifically inhibits RANKL and prevents its pairing with RANK and the resultant osteolysis and hence, has utilization in the management of GCT. However, it has little effect on the RANKL producing stromal cells, causing chances of recurrence as high as 15% after discontinuation of D-ab therapy [16]. Rapid recurrence of GCT after discontinuation of D-ab has been previously reported in the literature [1]. However, to the best of our knowledge, this is the first such case concerning the upper cervical spine. 

Although D-ab is increasingly being used as an adjuvant therapy for GCT, future research surrounding its utilization is required. The current literature does not define any endpoint in terms of duration for the use of D-ab while treating GCT. Tartrate-resistant acid phosphatase (TRACP) 5b, a bone resorption marker, is typically high in patients with GCT and has been shown to decrease drastically post-denosumab administration [17]. It might prove to be a good screening tool to check for recurrence after cessation of D-ab therapy. It is also not clear whether a re-administration of D-ab in case of recurrence can achieve the desired response due to the rapid progression of the tumor mass and symptoms. Possible association of prolonged use of D-ab with hypercalcemia, osteonecrosis of the jaw, and sarcoma also needs to be studied.

## Conclusions

Management of GCT of the cervical spine is challenging due to its local aggressiveness and proximity to critical neurovascular structures. Hence, the role of adjuvant therapy is vital in the treatment of upper cervical spine GCT due to the high chances of recurrence resulting from limited resection. D-ab, by inhibiting RANKL, prevents activation of osteoclasts and hence, acts as a potent adjuvant therapy for GCT. However, there is currently no consensus regarding dosing and duration of D-ab therapy after resection of GCT, and rapid recurrence of the tumor after its discontinuation can occur.
